# VIGILANCE, STRESS COPING, AND DISPARITIES IN METABOLIC HEALTH OVER THE LIFE COURSE

**DOI:** 10.1093/geroni/igac059.1638

**Published:** 2022-12-20

**Authors:** Viktoryia Kalesnikava, Michele Evans, Alan Zonderman, Briana Mezuk

**Affiliations:** University of Michigan, School of Public Health, Ann Arbor, Michigan, United States; National Institute on Aging, Bethesda, Maryland, United States; National Institute on Aging, National Institute of Health, Baltimore, Maryland, United States; University of Michigan, School of Public Health, Ann Arbor, Michigan, United States

## Abstract

Recent investigations of the mechanisms through which stress may impact cardiometabolic health point to the potential importance of cognitive tendencies (e.g., vigilance, coping strategies) in this relationship. This study examined the associations between vigilance and stress coping with metabolic risk, and whether these associations varied by race and life course socioeconomic status (SES). Data come from the Healthy Aging in Neighborhoods of Diversity across the Life Span (HANDLS) study, a cohort of urban adults (n=3720, baseline age 30-64 years; balanced by race, sex, age and household income). Vigilance was measured by the MacArthur Reactive Responding subscale; stress coping was measured by the Brief Cope scale. Metabolic risk was operationalized by metabolic syndrome z-scores (MetS-Z). Linear mixed models were used to examine longitudinal associations between cognitive tendencies and MetS-Z; interaction terms were used to examine effect modification by race and life course SES. Black participants reported higher adaptive coping; participants experiencing persistent poverty or downward mobility reported higher vigilance and avoidant coping and lower adaptive coping. Overall, vigilance was not associated with metabolic risk. Avoidant and adaptive coping were inversely associated with baseline MetS-Z and further varied by sociodemographic factors. Higher adaptive coping was associated with lower MetS-Z among White adults, while higher avoidant coping was associated with lower MetS-Z among Black adults. Lower stress coping were associated with higher MetS-Z among White participants with low lifecourse SES or downward socioeconomic mobility. In sum, stress coping may modulate the stress-health relationship, but these associations must be interpreted within the intersection of contextual factors.

